# Supplementary material for: Microfluidic multipoles theory and applications
Source: Nat Commun. 2019 Apr 16;10:1781. doi: 10.1038/s41467-019-09740-7 (PMC6467910; doi:10.1038/s41467-019-09740-7)
Supplement: Supplementary file 1 — Supplementary Information [file 41467_2019_9740_MOESM1_ESM.docx]

**Supplementary Information**

**Microfluidic multipoles theory and applications**

Goyette et al.

**Supplementary Table 1: Previously published multipolar devices and relation to the solutions obtained via conformal mapping of the advection-diffusion equation.**

| Probe type | Application | Reference | Solution |
| --- | --- | --- | --- |
| Dipole Probe | Tissue staining. Precise marking of surfaces | Juncker, David, Heinz Schmid, and Emmanuel Delamarche. "Multipurpose microfluidic probe." *Nature materials* 4.8 (2005): 622. | Transformation of dipole solution |
| Thermal Probe | Allows exposition of localized parts of a tissue to be exposed to different temperatures | Cors, Julien F., A. Stucki, and Govind V. Kaigala. "Hydrodynamic thermal confinement: creating thermo-chemical microenvironments on surfaces." *Chemical Communications* 52.88 (2016): 13035-13038. | Not applicable, as Peclet numbers are too low |
| Hydrodynamically confined microflow devices | Generation of uniform shear on open surfaces | Christ, Kevin V., and Kevin T. Turner. "Design of hydrodynamically confined microfluidics: controlling flow envelope and pressure." *Lab on a Chip* 11.8 (2011): 1491-1501. | Not applicable, our solution encompasses only point-like sources |
| Microfluidic Quadrupole | Adjustable concentration gradients | Qasaimeh, Mohammad A., Thomas Gervais, and David Juncker. "Microfluidic quadrupole and floating concentration gradient." *Nature communications* 2 (2011): 464. | Transformation of dipole solution for outline or separate quadrupole solution for the entire profile |
| Lateral Quadrupole | Adjustable concentration gradients | Brimmo, Ayoola T., and Mohammad A. Qasaimeh. "Microfluidic Probes and Quadrupoles: A new era of open microfluidics." *IEEE Nanotechnology Magazine* 11.1 (2017): 20-31. | Same as microfluidic quadrupole |
| Micro-Chemical Pen | Adjustable concentration gradient | Mao, Sifeng, et al. "Microchemical Pen: An Open Microreactor for Region‐Selective Surface Modification." *ChemPhysChem* 17.20 (2016): 3155-3159. | Transformation of dipole solution |
| Nested Dipoles | Allows flow pinching for more efficient use of chemicals during staining | Autebert, Julien, et al. "Hierarchical hydrodynamic flow confinement: efficient use and retrieval of chemicals for microscale chemistry on surfaces." *Langmuir* 30.12 (2014): 3640-3645. | Transformation of Choi et al’s numerical solution for the finite absorber^[[1]](#footnote-1)^ |
| Floating probe | Additional injection apertures allow control of the vertical position of the probe | Hitzbleck, Martina, et al. "The floating microfluidic probe: Distance control between probe and sample using hydrodynamic levitation." *Applied Physics Letters* 104.26 (2014): 263501. | Transformation of Choi et al’s numerical solution for the finite absorber |
| Dipole-array Probe | Sequential or simultaneous exposition of a tissue to different reagents in one experiment | Taylor, David P., et al. "Centimeter-Scale Surface Interactions Using Hydrodynamic Flow Confinements." *Langmuir* 32.41 (2016): 10537-10544. | Transformation of dipole solution |
| Channel injection in open chambers | Separate environment generation in open chambers | Olofsson, Jessica, et al. "A microfluidics approach to the problem of creating separate solution environments accessible from macroscopic volumes." *Analytical chemistry* 76.17 (2004): 4968-4976.  Ainla, Alar, Gavin Jeffries, and Aldo Jesorka. "Hydrodynamic flow confinement technology in microfluidic perfusion devices." *Micromachines* 3.2 (2012): 442-461.  Cheng, Jonathan W., et al. "An open-chamber flow-focusing device for focal stimulation of micropatterned cells." *Biomicrofluidics* 10.2 (2016): 024122. | Transformation of dipole solution |
| 2-gonal probe  Circular probe with injection in the middle | Precise staining of surfaces. The circular geometry ensures practically vertical concentration gradients | Oskooei, Ali, and Govind V. Kaigala. "Deep-Reaching Hydrodynamic Flow Confinement: Micrometer-Scale Liquid Localization for Open Substrates With Topographical Variations." *IEEE Transactions on Biomedical Engineering* 64.6 (2017): 1261-1269. | Transformation of dipole solution for the 2-gon  Circular probe is a limit case of polygonal probes and possesses an infinitely sharp gradient |

**Supplementary Note 1: Validity of the model for high Peclet numbers**

To generate our solution in streamline coordinates, we have used the solution for a semi-infinite absorber in an infinite plane. However, the problem in streamline coordinates possesses two axes of symmetry, one at $\psi=\pi$ and one at $\psi=\left( 1-\alpha\right)\pi$ (see supplementary table 2 and supplementary figure 1). In highly convective regime, the unbounded symmetry will still be valid for a certain region, as long as the symmetry axes are far enough from the wake. We will show that, for high Péclet numbers, the region in which this approximation breaks down is confined to an exponentially small region around the aspiration aperture. In practice, the region around the aspiration aperture is already excluded from our model since we our apertures are not point-like but are of finite size.

We will consider the axis of symmetry that is closest to the plate, which is, for $\alpha>2$ at $\psi=\pi$. (The other case can easily be done using a similar argument and reach the same conclusion but in practice we always use $\alpha>2$ as otherwise it is experimentally difficult to achieve correct confinement of the liquid). If the wake is far enough from the symmetry axis, we can assume that the unbounded space solution will be appropriate. We will consider that the wake is too close to the symmetry axis at the $\phi$ coordinate for which the concentration at $\psi=\pi$ predicted by the unbounded solution is 0.1 times the concentration of the plate. A similar development could be made for any other criterion (for example 0.01 times the concentration).


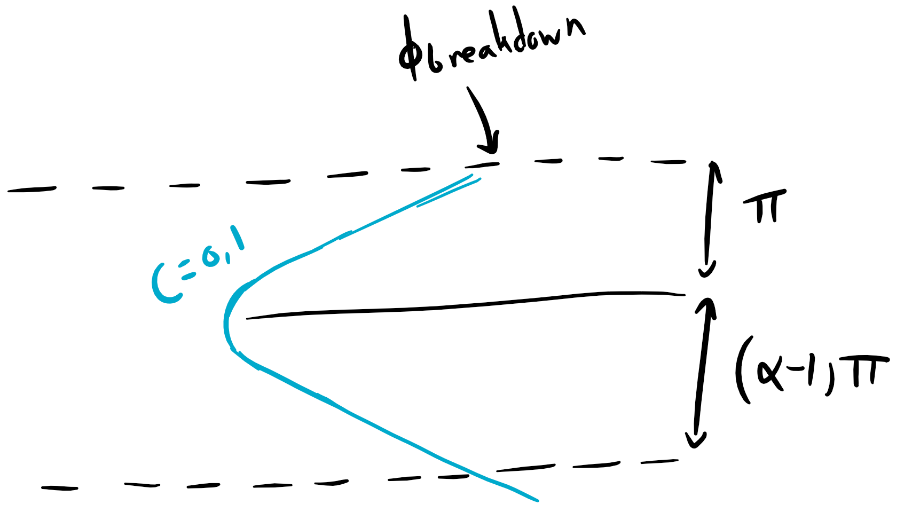


***Supplementary Figure 1 Axes of symmetry in the streamline coordinates***

We thus have

$$\mathrm{erfc} \left( \text{Im}\sqrt{\text{Pe}\left( \Phi-\Phi_{\mathrm{stag}} \right)} \right)=0.1$$

$$\text{Im}\sqrt{\left( \Phi-\Phi_{\mathrm{stag}} \right)}\approx\frac{1.163}{\sqrt{\text{Pe}}}$$

Taking $\omega=\sqrt{\left( \Phi-\Phi_{\mathrm{stag}} \right)}$, and labeling the real part of $\omega$ as $\omega_{\mathrm{real}}$

$$\omega=\omega_{\mathrm{real}}+\frac{1.163}{\sqrt{\text{Pe}}}i=\sqrt{\Phi-\Phi_{\mathrm{stag}}}$$

$$\Phi-\Phi_{\mathrm{stag}}=\left( \omega_{\mathrm{real}}+\frac{1.163}{\sqrt{\text{Pe}}}i \right)^{2}$$

$$\Phi-\Phi_{\mathrm{stag}}=\left( \omega_{\mathrm{real}}^{2}-\frac{{1.163}^{2}}{\text{Pe}} \right)+2\omega_{\mathrm{real}}\frac{1.163}{\sqrt{\text{Pe}}}i=\phi+\psi i$$

By taking $\psi=\pi$, we can isolate $\omega_{\mathrm{real}}$ and insert it in the expression for $\phi$ to find the position of the breakdown point. Thus, the unbounded solution is 0.1 times the plate concentration at $\psi=\pi$ for

$$\phi_{\mathrm{breakdown}}-\phi_{\mathrm{stag}}=\frac{\text{Pe} \pi^{2}}{4\cdot{1.163}^{2}}-\frac{{1.163}^{2}}{\text{Pe}}$$

For large enough Péclet numbers, we can consider only the term linear in Pe. We thus conclude that, in the streamline coordinate, the unbounded solution breaks down for $\phi-\phi_{\mathrm{stag}}>\frac{\pi^{2}}{4\cdot{1.163}^{2}}\text{Pe}$, provided Pe is large.

Let’s now see where this breakdown point maps in the $(x,y)$, dipole plane. Specifically, all values of $\phi$ over this breakdown value will map to the inside of a region around the aspiration aperture. We now want to see the size of this region.

We know that $\Phi$ is related to $z=x+y i$ by the complex potential, namely

$$\phi_{\mathrm{breakdown}}-\phi_{\mathrm{stag}}+\pi i=\log\left( z_{b} \right)-\alpha\log(z_{b}+1)$$

For high Péclet numbers, we know that this will map to a region close to $z=-1$, and can thus consider only the term $-\alpha log(z+1)$, which will be exponentially bigger than the term $\log(z)$

$$x_{b}=\text{real}(z_{b})=\exp\left( -\frac{1}{\alpha} \left( \phi_{\mathrm{breakdown}}-\phi_{\mathrm{stag}} \right) \right)-1$$

$$x_{b}+1=\exp\left( -\frac{\pi^{2}}{4\cdot{1.163}^{2}\cdot\alpha}\text{ Pe} \right)$$

We thus see that the distance between the breakdown point and the aspiration aperture (located at $x=-1)$) decreases as $exp(-\frac{\pi^{2}}{4\cdot{1.163}^{2}\cdot\alpha} \text{Pe})$. We plot this distance $r_{b}$ vs Pe in the following figure


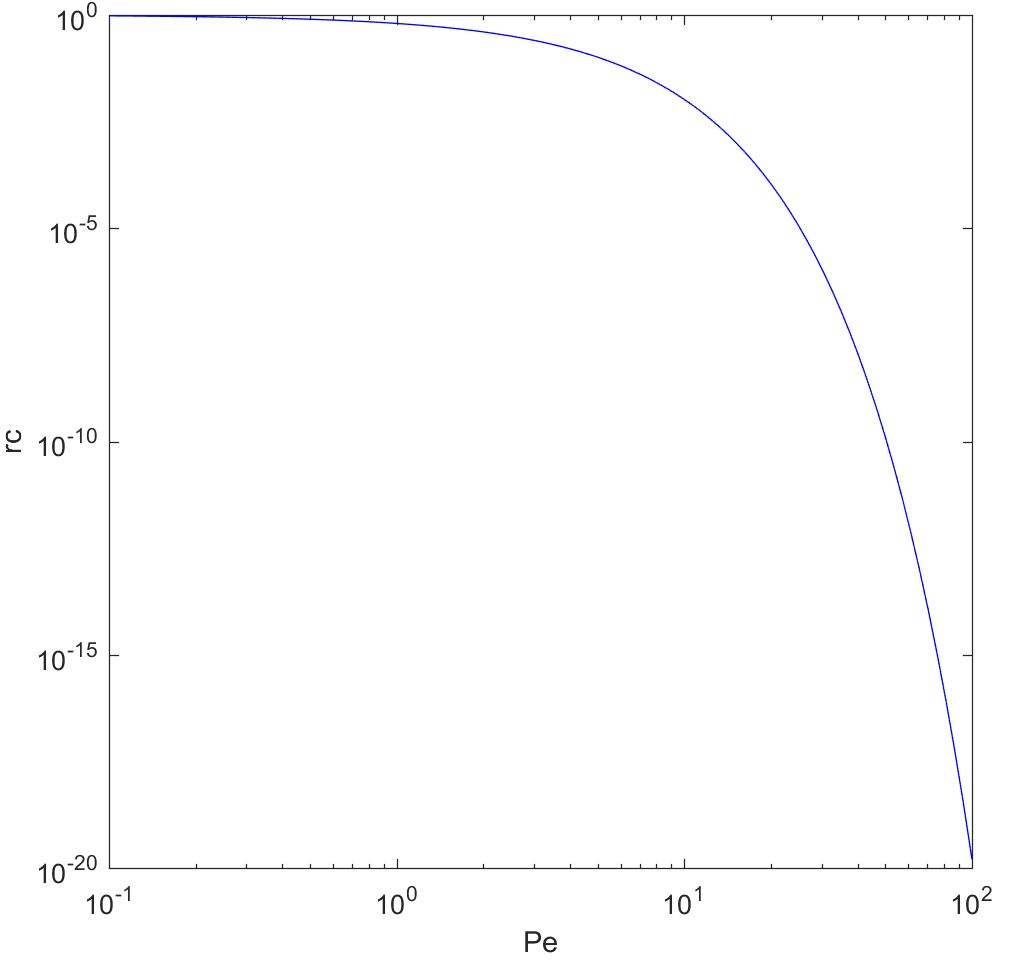


***Supplementary Figure 2 breakdown distance*** $\boldsymbol{r}_{\mathbf{c}}$ ***as a function of Péclet number*.** *For high Péclet numbers, this distance vanishes exponentially. Figure done for* $\alpha=4$

In a dipolar device with $\text{Pe}=10$, $\alpha=4$, the “breakdown region” in which the model is no longer accurate is confined to a distance of $0.01$ of the aspiration aperture. In practice, the aperture size is about a tenth of the distance between them, so the breakdown region is well under the apertures, a region in which the point-like sources and sink approximation already breaks down.

**Supplementary Table 2: Features of the dipole flow and their image in streamline coordinates**

| Z-plane coordinates | Streamline coordinates |
| --- | --- |
| Separating line  (See supplementary note 2 for expression) | $\phi\in]\phi_{\mathrm{stag}},\infty[$  $\psi=0$ |
| $x\in]-\infty,-1[$  $y=0$ | $\phi\in]-\infty,\infty[$  $\psi=\left( 1-\alpha\right)\pi$ |
| $x\in]-1,0[$  $y=0$ | $\phi\in]-\infty,\infty[$  $\psi=\pi$ |
| $x\in]0,x_{\mathrm{stag}}[$  $y=0$ | $\phi\in]-\infty,\phi_{\mathrm{stag}}[$  $\psi=0^{+}$ |
| $x\in]x_{\mathrm{stag}},\infty[$  $y=0$ | $\phi\in]-\infty,\phi_{\mathrm{stag}}[$  $\psi=0^{-}$ |

**Supplementary Note 2: Expression for the separating line in polar coordinates**

The solution for the concentration profile in a microfluidic dipole requires the identification of the separating line of concentration $c=\frac{1}{2}$, which determines the separating line between the dipole’s inner and outer regions. This line is the image of a semi-infinite segment of the real axis in the streamline domain and can thus be identified by looking for a change of sign in the imaginary part of the complex potential function. However, we can also derive a parametric expression for this separating line that is sometimes more convenient to use.

*
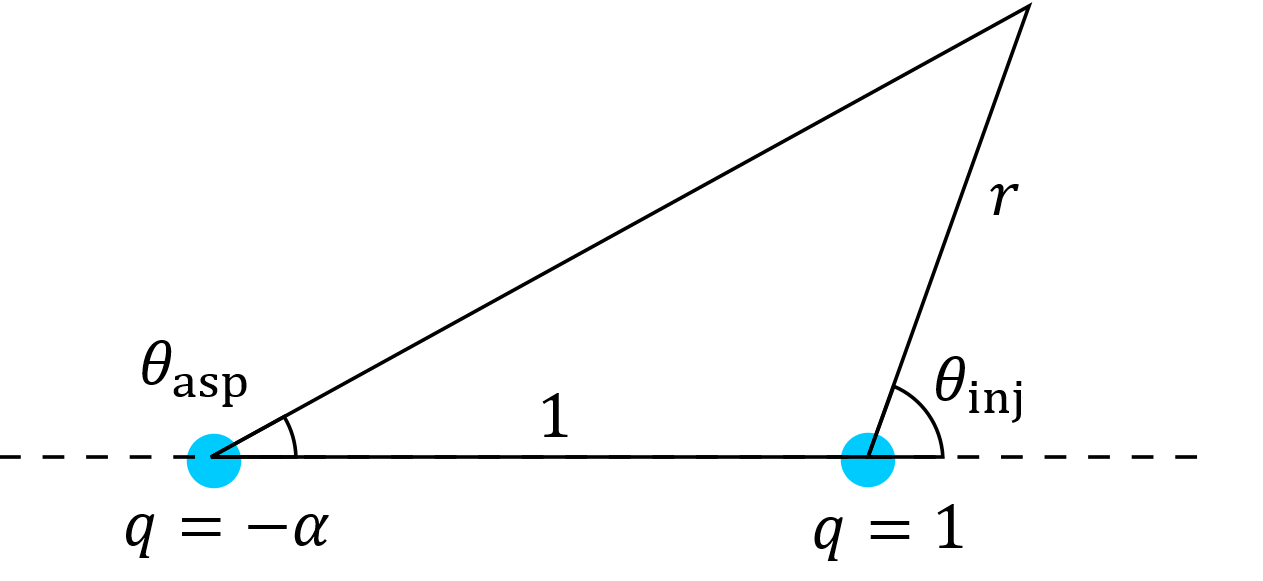
*

***Supplementary Figure 3 Defining the parametric expression for the separating line in polar coordinates***

The law of sines allows us to write

$$\frac{\sin{(\theta}_{\mathrm{asp}})}{r}=\frac{\sin(\pi-\theta_{\mathrm{inj}}-\theta_{\mathrm{asp}})}{1}=\sin(\theta_{\mathrm{inj}}-\theta_{\mathrm{asp}})$$

Which can be rewritten as

$$r= \frac{\sin(\theta_{\mathrm{asp}})}{\sin(\theta_{\mathrm{inj}}-\theta_{\mathrm{asp}})}$$

The stream function gives us

$$\psi=-\alpha\left( \theta_{\mathrm{asp}}+2\pi k_{1} \right)+\left( \theta_{\mathrm{inj}}+2\pi k_{2} \right)=k \pi$$

With $k_{1}, k_{2}, k\mathbb{\in Z}$

$$-\alpha\theta_{\mathrm{asp}}+\theta_{\mathrm{inj}}=(n_{1}+\alpha n_{2}) \pi$$

With $n_{1}=k-2 k_{2}$ and $n_{2}= 2 k_{1}$

We may substitute this expression in the expression for $r$ to obtain

$$r= \frac{\sin\left( \frac{1}{\alpha}\left( \theta_{\mathrm{inj}}-\left( n_{1}+\alpha n_{2} \right)\pi\right) \right)}{\sin\left( \left( 1-\frac{1}{\alpha} \right)\theta_{\mathrm{inj}}+\frac{1}{\alpha}\left( n_{1}+\alpha n_{2} \right)\pi\right)}$$

By taking the phase offset $n_{1}=n_{2}=0$, we obtain the simple expression

$$r= \frac{\sin\left( \frac{1}{\alpha}\theta_{\mathrm{inj}} \right)}{\sin\left( \left( 1-\frac{1}{\alpha} \right)\theta_{\mathrm{inj}} \right)}$$

Which, for $\theta_{\mathrm{inj}}\in]-\pi, \pi]$ defines the separating line of concentration $c=\frac{1}{2}$

**Supplementary Note 3: Other families of profiles, beyond the dipole solution**

We mention in the main text that an arbitrary placement of injection and aspiration apertures will in general not be reducible to a semi-infinite absorber (that is, the problem of convection-diffusion in streamline coordinates with concentration fixed on a semi-infinite strip of the real axis). Some important systems which cannot be reduced to a single semi-infinite absorber include the microfluidic quadrupole in which only a single aperture has a nonzero reagent concentration or the hierarchical microfluidic probe. In the case of such devices, the procedure outlined in section 1.1 can still be used, but the starting concentration profile is different from the semi-infinite absorber and corresponds to the inverse transform of the separating line in the respective geometry. In the case of an asymmetrical quadrupole, the contour of the footprint maps to two semi-infinite absorbers, while for the hierarchical probe, the contour maps to a finite absorber in streamline coordinates. The diffusion wake around those geometries in streamline coordinates do not possess a known analytical form, but asymptotic expressions valid at high Péclet number can be obtained, which allows us to retain simple expressions for the concentration profiles of these geometries at their operating parameters.

For the particular case of the quadrupole, the wake around a pair of semi-infinite absorbers can be approximated by the superposition of two wakes for single absorbers. At high enough Péclet numbers, the region where this approximation breaks down tends towards a limited region under the aspiration aperture, and thus does not impact our results. A concentration profiles obtained for the quadrupole is illustrated in Figure 3b. For the hierarchical probe, the solution for a finite absorber in a straight does not possess a simple analytical expression, but precise numerical schemes have been obtained (see Choi & al, 2004).

The way different starting profiles can be mapped to practical multipolar devices is illustrated in Supplementary Table 3. All profiles obtained by mapping the single semi-infinite absorber are exact. Solutions obtained from the pair of semi-infinite absorbers are asymptotic but remain extremely precise for any value of Pe over ~10, where the error stays confined under the apertures. Solutions obtained from Choi’s numerical approximation retain the same error as the original solution for the semi-infinite absorber.

Supplementary Table 1 contains a list of all previously published microfluidic probe geometries. All of them can be modelled using our method with the exception of Christ and Turner’s hydrodynamically confined microflow devices, for which the hypothesis of point-like apertures breaks down.

**Supplementary Table 3: Generating several probe families from basic solutions.** Left column, canonical solution groups in Boussinesq coordinates. Middle column: Basic elements from multipolar flow families. Right Column: examples of advanced applications achieved via conformal mapping from the basic multipolar elements (middle column). All theoretical results stem from the results derived in this paper. References in table elements indicates multipolar embodiments realized experimentally and published. Non-referenced table elements indicate those geometries are new and not hitherto realized experimentally.

^
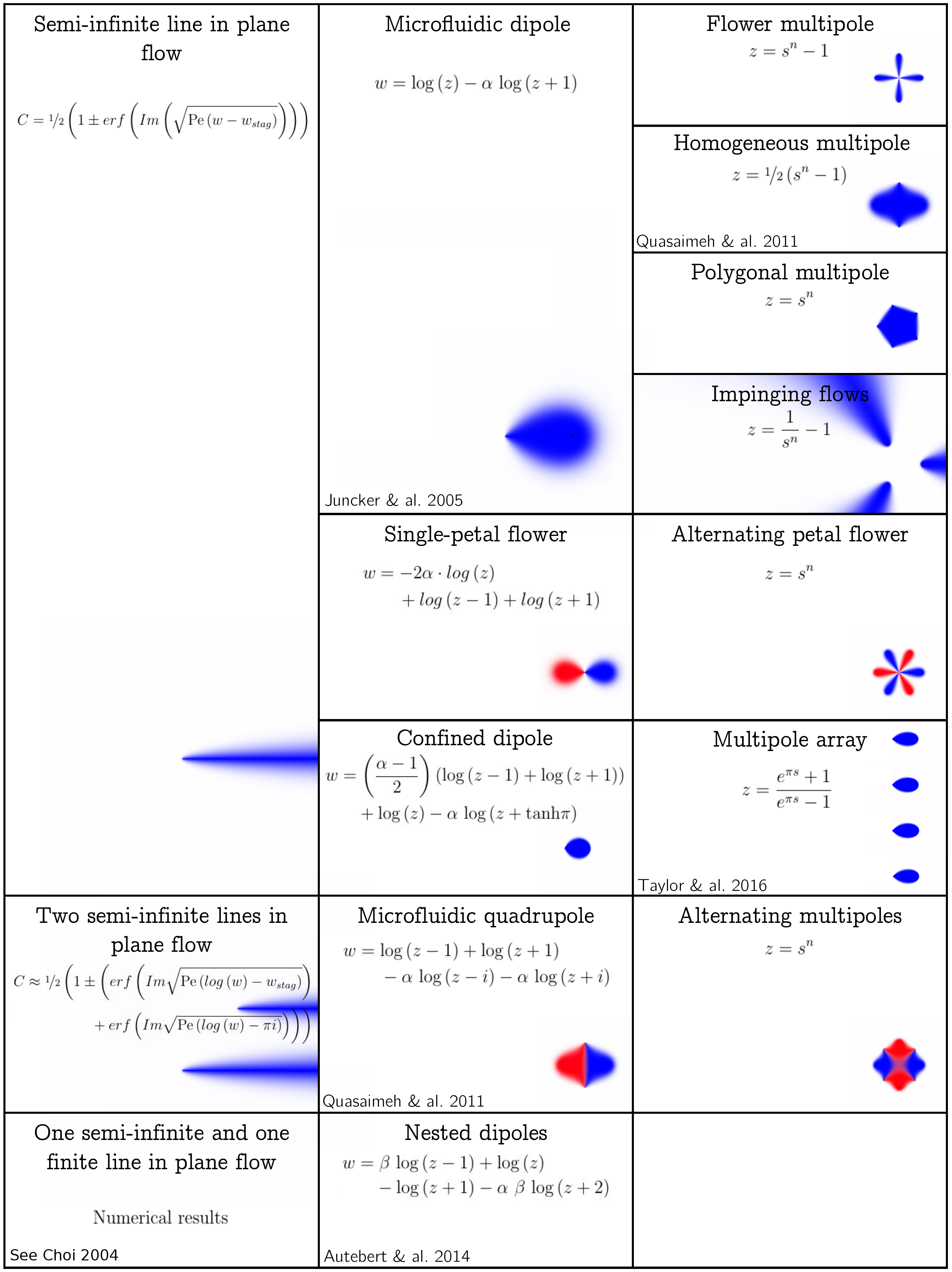
^


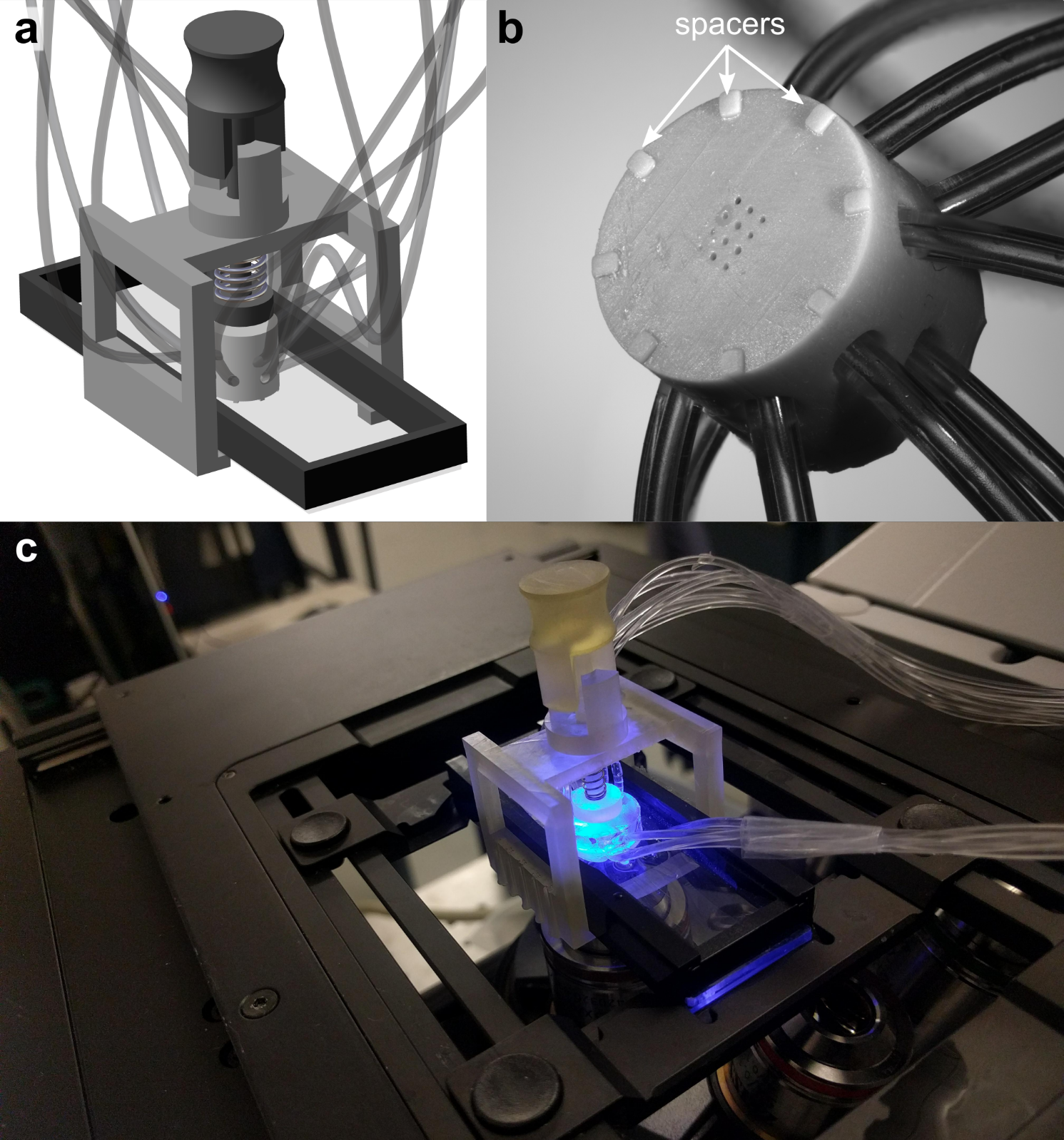
 ***Supplementary Figure 4 MFM auto-alignment setup.*** *(a) CAD of the MFM positioning system. (b) Closeup of an MFM showing the auto-alignment pillars. (c) Photo of the 3D printed MFM positioning system.*
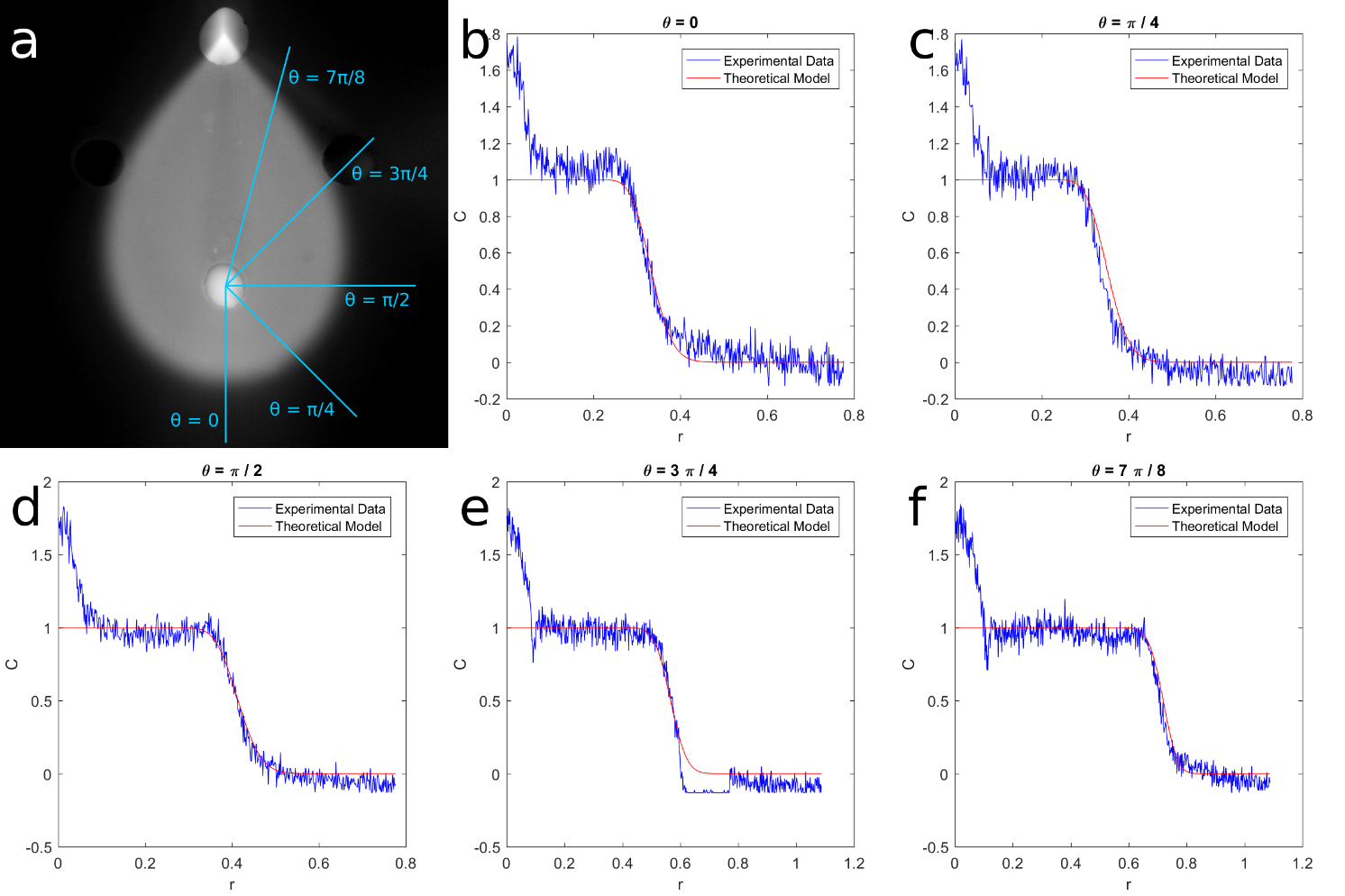


***Supplementary Figure 5 Comparison between theoretical model and experimental results for different cross-sections of a microfluidic dipole.*** *How each cross-section maps to the experimental image is illustrated in a), while b)-e) show the comparison of experimental and theoretical results. It is to be noted that the discrepancy in subfigure e) is due to the presence of another aperture on the multipole, which hides a part of the concentration gradient (visible in subfigure a). Both axis of the graphs b) to f) are in adimensional units. Scale bar: 300 µm.*


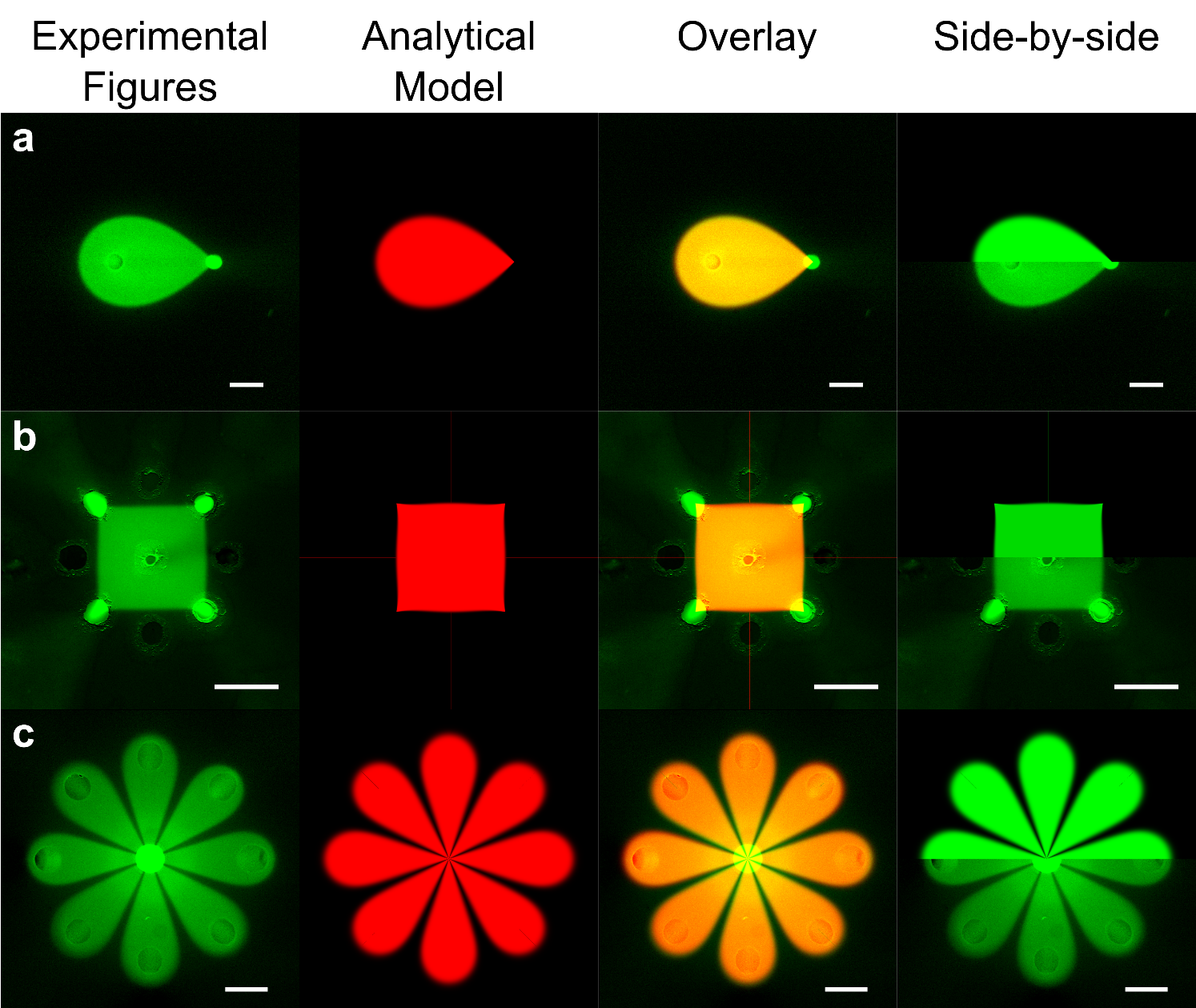


***Supplementary Figure 6 Comparison between experimental figures and the analytical model.*** *For each configuration, an experimental figure, an analytical model figure, an overlay between the experimental and analytical figure and a side-by-side comparison are shown. (a) Microfluidic dipole, (b) Polygonal multipole, (c) 8-petal axisymmetric “flower” multipole. Scale bars: 500 µm.*

**Supplementary Note 4: Fixed width rMFM α modulation**

The advection-diffusion solution of a rMFM with only one aperture turned on is the microfluidic dipole solution. When a second aperture is added, advection diffusion of a petal follows a similar solution, the dipole solution, but compressed in half the complex plane. This result in a compressed petal width. The same effect applies to every number of apertures. The advection-diffusion in the petal of an 8-petals rMFM follow the dipole solution but compressed in one eight of the complex plane. Using the exact flow model, α can be modulated to compensate the width compression for each number of petals turned on. Using such method, the width can be kept constant. Supplementary Table 4 present the alpha correction for 1 to 8 petals working for α_8_ =2.

**Supplementary Table 4: α correction for a constant pattern width using the rMFM**

| Nb pumps on | α Correction Factor | αeff (example for α=2) | Qaps (example for Qinj =0.2µL/s) |
| --- | --- | --- | --- |
|  |  | $\alpha_{eff}= \alpha_{8}\cdot correction factor$ | ${Q_{asp}=(Nb pumps)\alpha}_{eff}Q_{inj}$ |
| 8 | 1.00 | 2.00 | 3.20 |
| 7 | 1.06 | 2.12 | 2.97 |
| 6 | 1.15 | 3.00 | 3.60 |
| 5 | 1.25 | 2.50 | 2.50 |
| 4 | 1.42 | 4.00 | 3.20 |
| 3 | 1.76 | 3.52 | 2.11 |
| 2 | 2.44 | 5.00 | 2.00 |
| 1 | 4.72 | 9.44 | 1.89 |


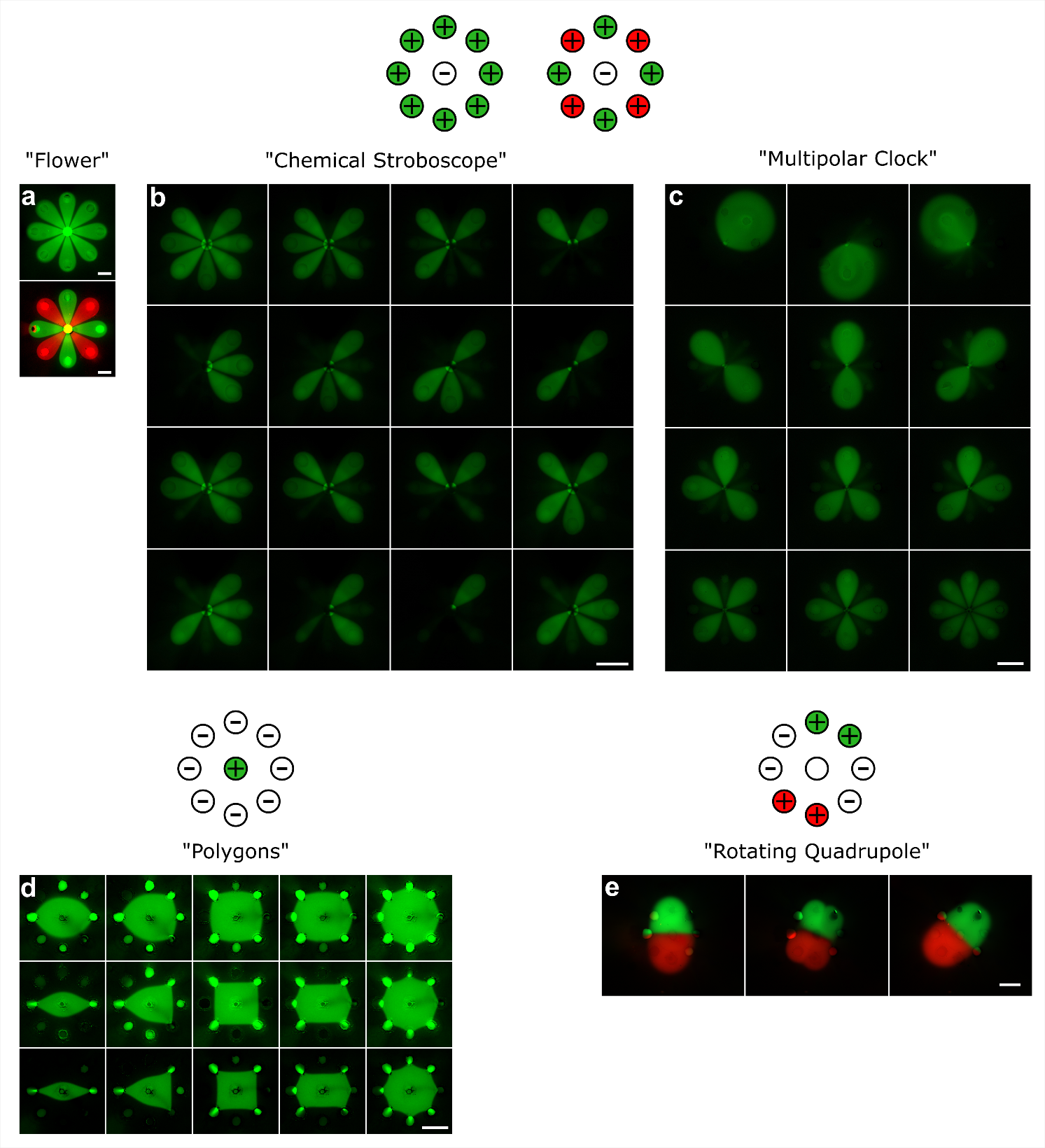


***Supplementary Figure 7****Some of the injection/aspiration configuration that can be made with a rMFM, and some of the patterns that can be made with each configuration. All images from a set are taken in a single experiment. a) Flower, b) Chemical Stroboscope (see Supplementary Video 1), c) Multipolar Clock (see Supplementary Video 2), d) Polygon (see Supplementary Video 3), e) Rotation Quadrupole. All scale bars are 500 µm.*


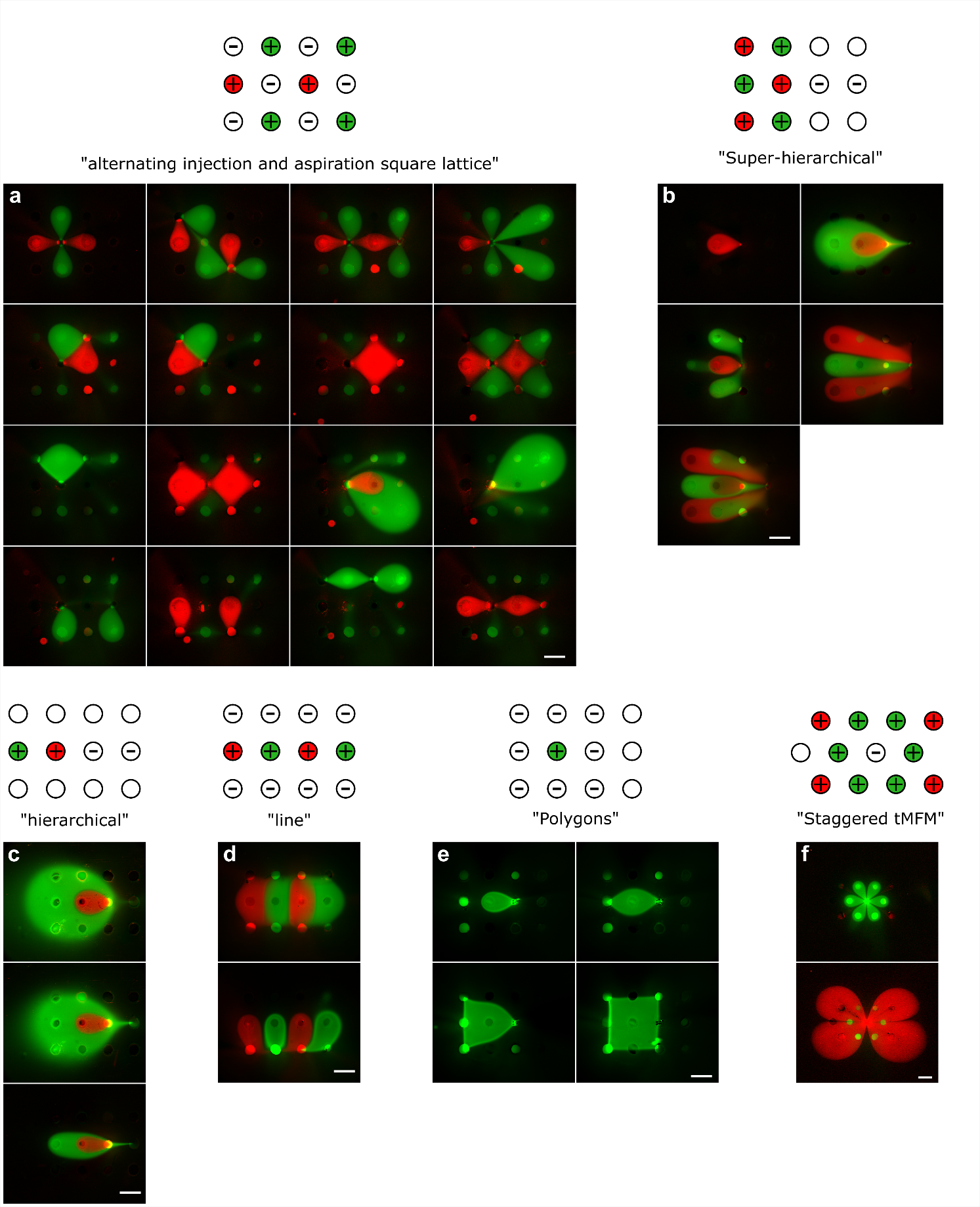


***Supplementary Figure 8*** *Some of the injection/aspiration configuration that can be made with a 12 apertures tMFM, and some of the pattern that can be made with each configuration. All images from a set are taken in a single experiment. a) Alternating square lattice (see Supplementary Video 4), b) Super-hierarchical, c) hierarchical, d) line, e) Polygons, f) Staggered tMFM. All scale bars are 500 µm.*

*
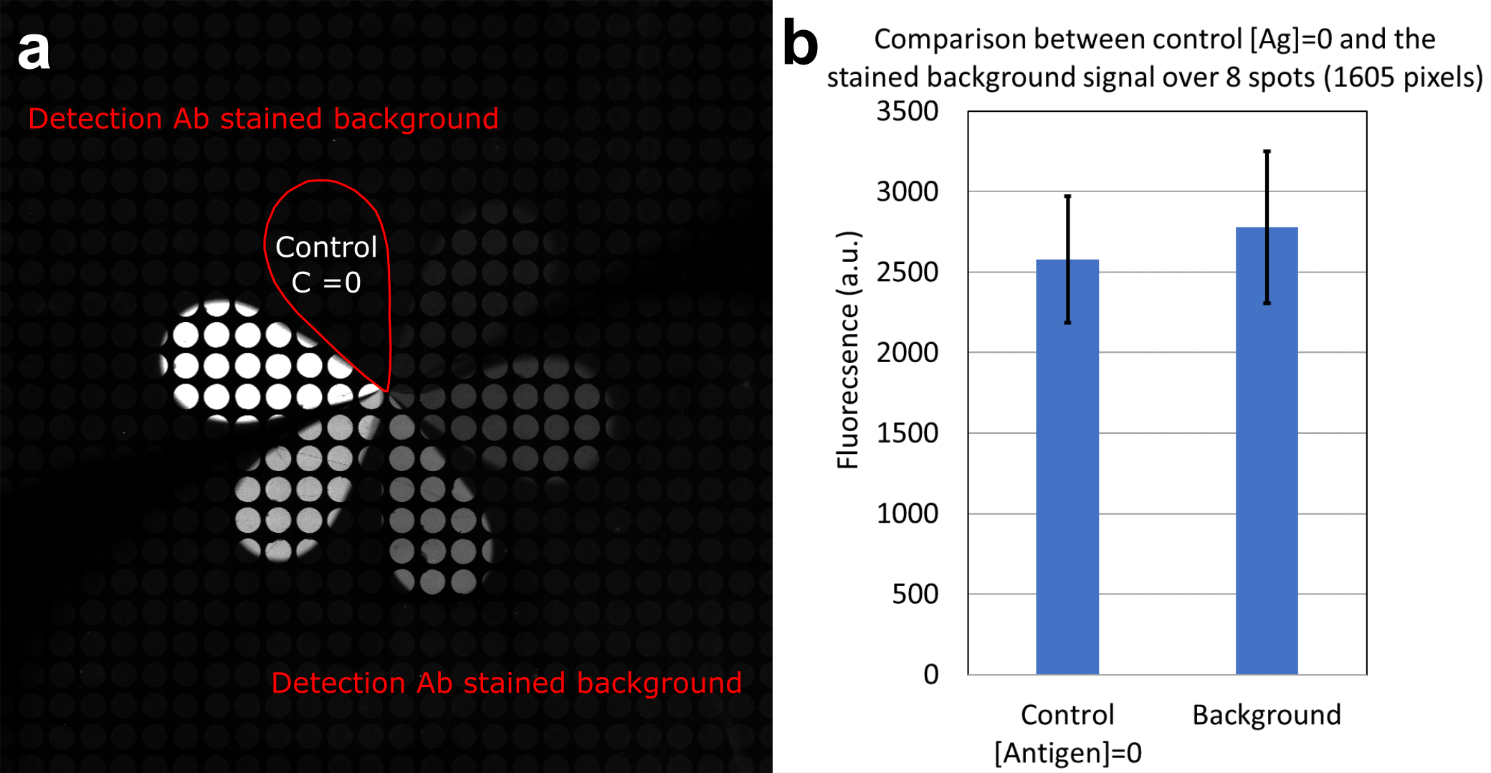
****Supplementary Figure 9*** *a) Fluorescent micrograph showing that the result of an immunoassay experiment with one confinement area with a null concentration of antigen. b) Graph showing that the fluorescent level of a confinement area with a null concentration of antigen and the detection Ab stained background have a similar fluorescent level. Measurement made on 8 spots and 1605 pixels. Other experiments showed similar results.*

**Supplementary Note 5: Using experimental or numerical results as source images**

We have presented the use of conformal maps to obtain a concentration profile for MFM devices by transforming a known analytical solution in a simpler geometry. This can be achieved because of the conformal invariance of the Hele-Shaw and 2D convection-diffusion equations. However, nothing prevents us to use the same conformal maps in situations where analytical solutions are not known. In fact, any initial concentration profile, be they analytical expressions, CFD results or experimental images, can be transformed to generate new concentration profiles. This technique is very useful when generalizing devices for which a complete analytical model is not known, as it still gives us the full power of conformal maps for deriving new symmetrical configurations.

As an example, we show in Supplementary Figure 8 how we can predict the same concentration profiles using different source images.


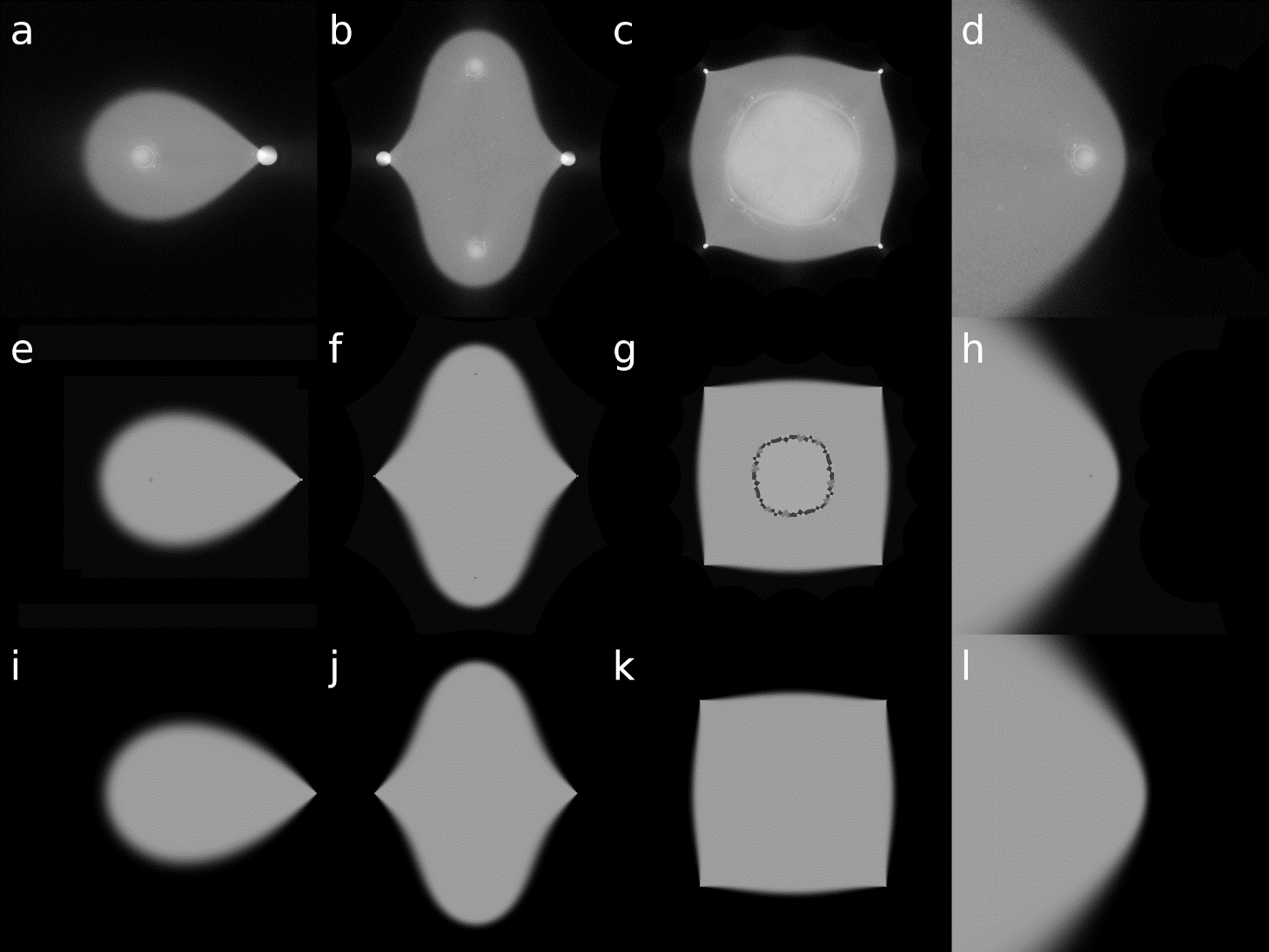
 ***Supplementary Figure 10*** *Using three different initial solutions to obtain concentration profiles in symmetrical devices. Top row: an experimental image of a dipole probe (a) is transformed to obtain the concentration profile in a quadrupole (b), a polygonal MFM (c) and impinging flows in a chamber (d). Second row: the same procedure is applied to an initial image obtained through a finite elements calculation (e) to obtain profiles for the three new geometries (f, g, h). The bottom row consists of the analytical dipole solution (i) transformed to the quadrupole, polygon and impinging flows (j, k, l). It can be seen that all three source images give equally valid results.*

1. Choi J, Margetis D, Squires TM, Bazant MZ, « Steady advection-diffusion around finite absorbers in 2D potential flow”, *J. Fluid. Mech*., 536, 2005, 155-184. [↑](#footnote-ref-1)
