# Supplementary material for: Microfluidic multipoles theory and applications
Source: Nat Commun. 2019 Apr 16;10:1781. doi: 10.1038/s41467-019-09740-7 (PMC6467910; doi:10.1038/s41467-019-09740-7)
Supplement: Supplementary file 3 — Description of Additional Supplementary Files [file 41467_2019_9740_MOESM3_ESM.pdf]

Description of Additional Supplementary Files

Supplementary Movie 1: Rotationally-symmetric MFM used as a chemical stroboscope. Each confinement area is turned on and off with a different duty-cycle and frequency, in a pulse with modulation fashion. This video is related to Figure 4a, b. Video speed: accelerated to 3x. Injection flow rate = 200nL/s

Supplementary Movie 2: Rotationally-symmetric MFM used in the “flower” configuration. The injections are modulated to form a microfluidic clock. This video shows many different possible fluid configurations using a rMFM. Injection flow rate = 200nL/s.

Supplementary Movie 3: Rotationally-symmetric MFM used in the “polygon” configuration. The aspirations are modulated to form a microfluidic clock. This video shows another set of possible configurations using a rMFM. Injection flow rate = 2µL/s.

Supplementary Movie 4: Translationally-symmetric MFM used to make 28 different patterns in less than 2 minutes by modulating the injections and the aspirations. This is the video version of Figure 4c. Video speed: accelerated to 2x.

Supplementary Dataset 1 - dipole MFM: A 3D printable .stl file containing the dipole MFM design. This dipole design was used for Figure 3c.

Supplementary Dataset 2 - quadrupole MFM: A 3D printable .stl file containing the quadrupole MFM design. This quadrupole design was used for Figure 1f and Figure 3d.

Supplementary Dataset 3 – 8-channel rMFM (small): A 3D printable .stl file containing the 8-channel rMFM design. This is one of the two version of rMFM used for this article. This design was used for Figure 4a.

Supplementary Dataset 4 – 8-channel rMFM (large): A 3D printable .stl file containing the larger version of a 8-channel rMFM design. This is one of the two version of rMFM used for this article. This design was used for Figure 1g and Figure 3g.

Supplementary Dataset 5 – 12-channel tMFM: A 3D printable .stl file containing the 12-channel tMFM design. This design was used for Figure 4c.

Supplementary Dataset 6 - staggered 12-channel tMFM: A 3D printable .stl file containing the staggered 12-channel tMFM design. This design was used for the immunoassay experiments (Figure 5
